# Optimal hypofractionated conformal radiotherapy for large brain metastases in patients with high risk factors: a single-institutional prospective study

**DOI:** 10.1186/s13014-014-0231-5

**Published:** 2014-10-17

**Authors:** Hiroshi K Inoue, Hiro Sato, Yoshiyuki Suzuki, Jun-ichi Saitoh, Shin-ei Noda, Ken-ichi Seto, Kota Torikai, Hideyuki Sakurai, Takashi Nakano

**Affiliations:** Cyber Center, Kanto Neurosurgical Hospital, 1120 Dai, Kumagaya, Saitama 360-0804 Japan; Neurosurgery and Radiation Oncology, Institute of Neural Organization, 1120 Dai, Kumagaya, Saitama 360-0804 Japan; Department of Radiation Oncology, School of Medicine, Fukushima Medical University, 1 Hikarigaoka, Fukushima City, Fukushima 960-1295 Japan; Department of Radiation Oncology, Gunma University Graduate School of Medicine, 3-39-22 Showa-machi, Maebashi, Gunma 371-8511 Japan; Gunma University Heavy-ion Medical Research Center, 3-39-22 Showa-machi, Maebashi, Gunma 371-8511 Japan; Proton Medical Research Center, University of Tsukuba, 2-1-1 Amakubo, Tsukuba, Ibaraki 365-8576 Japan

**Keywords:** Large brain metastases, Hypofractionated conformal radiotherapy, Multi-session radiosurgery, Prediction of complications, Radiation necrosis, Brain edema, Optimal dose and fraction, V14

## Abstract

**Background:**

A single-institutional prospective study of optimal hypofractionated conformal radiotherapy for large brain metastases with high risk factors was performed based on the risk prediction of radiation-related complications.

**Methods:**

Eighty-eight patients with large brain metastases ≥10 cm^3^ in critical areas treated from January 2010 to February 2014 using the CyberKnife were evaluated. The optimal dose and number of fractions were determined based on the surrounding brain volume circumscribed with a single dose equivalent (SDE) of 14 Gy (V14) to be less than 7 cm^3^ for individual lesions. Univariate and multivariate analyses were conducted.

**Results:**

As a result of optimal treatment, 92 tumors ranging from 10 to 74.6 cm^3^ (median, 16.2 cm^3^) in volume were treated with a median prescribed isodose of 57% and a median fraction number of five. In order to compare the results according to the tumor volume, the tumors were divided into the following three groups: 1) 10–19.9 cm^3^, 2) 20–29.9 cm^3^ and 3) ≥30 cm^3^. The lesions were treated with a median prescribed isodose of 57%, 56% and 55%, respectively, and the median fraction number was five in all three groups. However, all tumors ≥20 cm^3^ were treated with ≥ five fractions. The median SDE of the maximum dose in the three groups was 47.2 Gy, 48.5 Gy and 46.5 Gy, respectively. Local tumor control was obtained in 90.2% of the patients, and the median survival was nine months, with a median follow-up period of seven months (range, 3-41 months). There were no significant differences in the survival rates among the three groups. Six tumors exhibited marginal recurrence 7-36 months after treatment. Ten patients developed symptomatic brain edema or recurrence of pre-existing edema, seven of whom required osmo-steroid therapy. No patients developed radiation necrosis requiring surgical resection.

**Conclusion:**

Our findings demonstrate that the administration of optimal hypofractionated conformal radiotherapy based on the dose-volume prediction of complications (risk line for hypofractionation), as well as Kjellberg’s necrosis risk line used in single-session radiosurgery, is effective and safe for large brain metastases or other lesions in critical areas.

## Background

Surgical removal is the gold standard therapy and is essential for treating large brain metastases causing progressive symptoms due to increased intracranial pressure, as the symptoms improve immediately after surgery. However, surgical removal carries a risk of causing neurological deficits after dissecting critical areas, especially in cases of tumors situated deep within the white matter. Surgery also requires hospitalization for at least one week, with higher medical expenses than that observed for radiosurgery in this country. In addition, there are many patients with general risks for surgery, as well as those who refuse surgical procedures, due to having primary malignancies and/or a poor performance status.

Whole brain radiotherapy (WBRT) and chemotherapy are not adequate to control large brain metastases, and radiosurgery is an important therapeutic tool for treating brain metastases in multiple clinical settings. Moreover, radiosurgery is increasingly being used as a primary treatment modality in an attempt to prevent disturbances in the neurocognitive function after WBRT [1]. However, single-session radiosurgery is also inadequate for managing large brain metastases due to dose limitations resulting from the need to prevent adverse effects on the surrounding structures, such as the optic pathway, internal capsule and brainstem [2].

Hypofractionated radiotherapy appears to be beneficial in case of metastases not causing clinical signs of impending cerebral herniation, and its use is supported by the findings of previously published series employing varying radiation dose and fractionation schedules [3–7]. However, the optimal dose and number of fractions have yet to be established [8], and the exact incidence of adverse effects on the surrounding brain is unclear in patients with tumors with high risk factors, such as a large size or location in a critical area. Therefore, in order to determine the optimal dose and fractionation schedule, dose escalation following low-dose treatment is required.

We previously reported that the brain volume circumscribed with a single dose equivalent (SDE) of 14 Gy (V14) is an indicator of radiation necrosis [9] and that the incidence of radiation-related complications after hypofractionated conformal radiotherapy is best predicted according to the dose-volume relationship using the SDE of the maximum dose and V14 [10]. In January 2010, we initiated a prospective study of optimal hypofractionated conformal radiotherapy based on risk prediction in order to avoid radiation necrosis after hypofractionation treatment for large brain metastases in addition to the Kjellberg’s necrosis risk line used in single-session radiosurgery [11].

This report presents the results of our single-institution prospective study of optimal hypofractionated conformal radiotherapy performed in this institute as useful treatment for patients with large brain metastases in critical areas and/or those with general risks for surgery.

## Methods

All patients provided their written informed consent prior to the procedure with institutional ethics committee approval. Ninety-seven patients with large brain metastases measuring 10 cm^3^ in volume or more were treated with optimal hypofractionated radiotherapy based on the dose-volume prediction of complications in order to avoid radiation necrosis and subsequently followed more than three months at Kanto Neurosurgical Hospital between January 2010 and February 2014. An evaluation of the rates of local tumor control, overall survival and complications was performed as a prospective single-institutional analysis.

### Definition of variables and end points

The treatment dose is expressed as the marginal dose used in hypofractionated conformal radiotherapy. The maximum dose was automatically obtained from the marginal dose and the prescribed isodose delivered to the lesion margin. A complication was defined as neurological impairment (either the development of a new deficit or the significant deterioration of a preexisting or recurrent deficit) with a change on either computerized tomography scans or magnetic resonance imaging studies. Clinical follow-up was considered to have stopped at the time of the most recent report from the patient and/or a representative or the time of death.

### Inclusion and exclusion criteria

Ninety-seven patients followed for more than three months after treatment were found to be eligible for inclusion in this study. Nine patients (9.3%) were excluded from the analysis due to a lack of available imaging findings after treatment. All other patients (88) were included in the analysis.

### Patient characteristics

The median age of the patients was 64 years; 42 patients (47.7%) were 65 years of age or older. The primary cancers were located in the lung, breast, gastrointestinal tract, ovary, kidney, thyroid, larynx, uterus, or other regions (liver, testis, etc.). The tumors (n =92) treated according to the hypofractionation protocol were situated in the frontal lobe (close to the optic pathway, Broca’s area or motor cortex), parietal lobe (sensory cortex or dominant angular cortex), temporal lobe (close to the optic pathway or Wernicke’s area), occipital lobe (visual cortex), thalamus, basal ganglia or cerebellum close to the brainstem. The median Karnofsky Performance status (KPS) score was 70, and 34 patients (38.6%) had a KPS score of less than 70. The initial tumor volume was measured using the MultiPlan (Accuray, Sunnyvale, CA) software program, which determines the treatment volume based on the findings of enhanced T1-weighted magnetic resonance imaging (MRI). The median tumor volume of 92 lesions was 16.2 cm^3^. Forty-nine tumors (53.3%) were larger than 15 cm^3^ (more than 3 cm in diameter). Thirteen tumors measured 30 cm^3^ or more (4 cm in diameter) up to 74.6 cm^3^. Table 1 shows the patient characteristics.

**Table 1:** **Pretreatment characteristics of the 88 patients with large brain metastases in critical areas**

Number of patients	88	Location of tumor	92 lesions
Median age (range)	64 (33–93)	Cerebral hemisphere	61
Age ≥65	42	Frontal	27
Age <65	46	Parietal	10
Sex		Temporal	13
Male	42	Occipital	21
Female	46	Thalamus, basal ganglia	4
Primary cancer		Cerebellum	17
Lung	41	Median KPS score	70 (50–100)
Breast	23	KPS ≥70	54
Gastro-intestinal tract	9	KPS <70	34
Ovary	4	Tumor volume, median (cm^3^)	16.2
Kidney	3	≥ 30.0	13
Thyroid	2	20.0-29.9	18
Larynx	2	10.0-19.9	61
Uterus	2	Image follow-up period (months)	
Others	6	Median	7
Multiple vs single		Range	1-37
Multiple metastases	45	Survival period (months)	
Single metastases	43	Median	9
Metastases to other organs	50	Range	3-41

### Prescribed marginal and SDE of the maximum dose

The maximum dose was calculated based on the marginal dose and prescribed isodose. For the purpose of the dose-volume analysis, the maximum dose in three- to ten-fraction treatment was converted to the SDE using the equation reported by Park et al. and Eaton et al. [8,12], as previously reported [10].

### Optimal hypofractionated conformal radiotherapy

Hypofractionated conformal radiotherapy was administered under CT and MRI guidance as previously reported [9]. When setting a dose and fraction schedule as the first plan, a marginal dose of 27–30 Gy in three fractions was intended to use to treat tumors measuring 10–19.9 cm^3^. A marginal dose of 31–35 Gy in five fractions and a marginal dose of 35–42 Gy in eight to 10 fractions were intended to use to treat tumors measuring 20–29.9 cm^3^ and tumors measuring 30 cm^3^ or more, respectively. The isodose volume of the surrounding brain (excluding the GTV) circumscribed with an SDE of 14 Gy (V14), as well as the tumor volume, was measured using the MultiPlan software program for the G4 system (Accuray, Sunnyvale, CA) and recorded in each patient in order to obtain the optimal dose and fractionation schedule. The SDE of 14 Gy used in three- to ten-fraction treatment was 23.1 to 38.4 Gy according to Timmerman’s values [13], as previously reported [10]. The V14 of each tumor was maintained at less than 7 cm^3^ in order to prevent radiation necrosis for optimal treatment based on the dose-volume prediction of complications [10]. If the V14 value was more than 7 cm^3^ in the first dose-plan, it was lowered to less than 7 cm^3^ using a decreased marginal isodose or dose or an increased number of fractions in the revised or re-revised dose-plan, maintaining an effective marginal dose (SDE: 18–20 Gy) on the target.

### Follow-up evaluations and complications

Changes in the patients’ neurological symptoms, such as paresis, sensory disturbances, aphasia or visual disturbances, were examined after treatment. Serial imaging studies (MRI or CT) were requested six weeks after treatment and every two to three months thereafter. Symptomatic brain edema was identified in association with neurological deterioration and imaging changes in the concomitant perifocal regions after treatment. The tumor response was divided into three groups, reduced (a decrease in the tumor volume of >15%), stable (a change in the tumor volume within ±15%) or enlarged (an increase in the tumor volume of >15%), in order to compare the response with that observed in former reports of large brain metastases [9,10]. The incidence of complications was examined in relation to the V14 of the surrounding brain.

### Statistical analysis

Univariate and multivariate analyses were conducted using logistic regression and Cox hazard models. Differences between the groups were evaluated using Student’s *t*-test. Overall survival was estimated according to the Kaplan-Meier method and examined for significance using the log-rank and generalized Wilcoxon tests. All analyses employed the conventional p < 0.05 level of significance*.*

## Results

Nineteen patients received osmo-steroid therapy during hypofractionation treatment for symptoms of perifocal edema and/or the further oral administration of steroids depending on the presence of other symptoms at the outpatient clinic.

### Follow-up evaluations

No new neurological deficits from direct damage to the optic pathway, brainstem or functional areas were noted, although symptoms recurred or appeared in 10 patients due to adverse effects (brain edema) on the surrounding brain.

### Treatment-related variables of optimal hypofractionation

The prescribed isodoses ranged from 50% to 70% (median, 57%) for the target. The SDE of the maximum dose ranged from 36.8 to 61.9 Gy (median, 47.4 Gy) delivered in three to 10 fractions (median, five). Twenty-one tumors were treated with more than five fractions. The median V14 value was 5.0 cm^3^ (Table 2). The results are shown for each of the three groups divided according to the tumor volume in Table 3. The prescribed isodose declined according to the tumor volume, although no significant differences were found among the three groups. Large tumors measuring 20 cm^3^ or more were treated with five fractions or more in order to maintain a V14 of less than 7 cm^3^. A large number of fractions (more than five) was used, even in tumors measuring less than 20 cm^3^, in order to decrease the V14 to less than 3 cm^3^ or 1 cm^3^ for tumors in critical areas, such as the motor cortex, basal ganglia, thalamus or pyramidal tract (concerning normal tissue dose constraints), or those associated with extensive brain edema. There were no significant differences in the SDE of the maximum dose or V14 values among the three groups.

**Table 2:** **Treatment-related variables of the 92 large brain metastases in the 88 patients**

Prescribed isodose (%)	
Median	57
Range	50-70
Fraction number	
Median	5
Range	3 – 10
Lesion treated with 3 fraction	14
4 fraction	2
5 fraction	55
6 fraction	7
8 fraction	12
10 fraction	2
Maximum dose (single fraction equivalent dose, Gy)	
Median	47.4
Range	36.8-61.9
V14 (cm^3^)	
Median	5
Range	0.3-6.9

**Table 3:** **Results of optimal hypofractionation in the three groups divided according to the tumor volumes**

Tumor volume (cm^3^) (median)	10–19.9 (13.4)	20–29.9 (23.8)	30–74.6 (37.5)
Median isodose (%) (range)	57 (50–70)	56 (51–64)	55 (51–64)
Median fraction Number (range)	5 (3–10)	5 (5–10)	5 (5–8)
Median SDE of the max. dose (Gy) (range)	47.2 (36.8-61.9)	48.5 (38.1-61.5)	46.5 (39.7-56.0)
Median V14 (cm^3^)	5.1	4.4	5.2
(range)	(0.3-6.9)	(0.7-6.1)	(0.4-6.1)
Median KPS	70	70	65
(range)	(50–100)	(50–80)	(50–90)
Tumor response			
Reduced (87)	58	17	12
Stable (2)	0	1	1
Enlarged (3)	3	0	0
Marginal recurrences (6)	4/61 (6.6%)	2/18 (11.1%)	0/13 (0%)
Complications (10) (brain edema)	5/61 (8.2%)	4/18 (22.2%)	1/13 (7.7%)

### Tumor response, local control and overall survival after treatment

**Figure 1:**
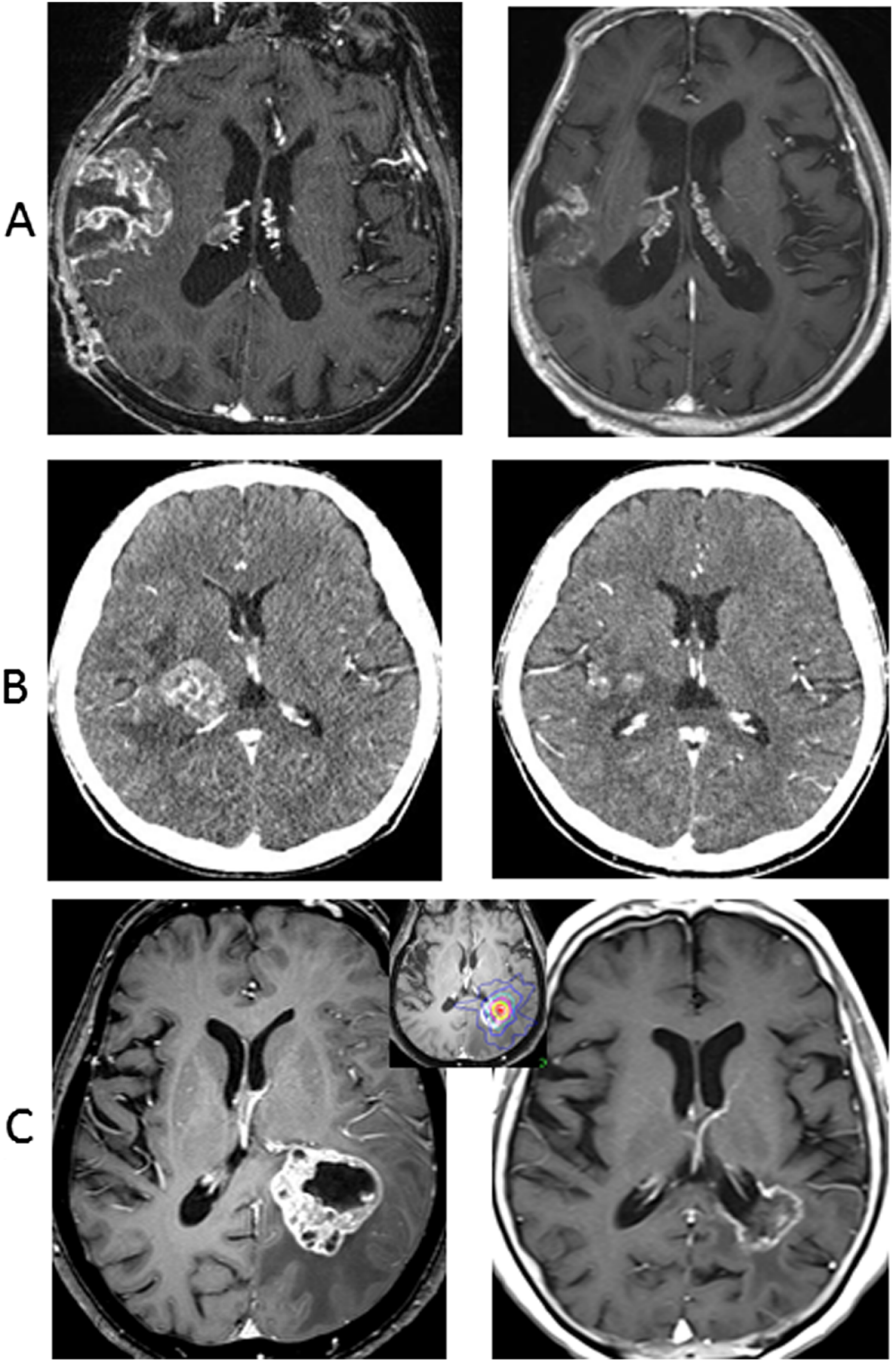
**Tumor regression after optimal hypofractionated conformal radiotherapy. A**: Gd-enhanced T1-weighted MR images. Lung cancer brain metastasis in a 73-year-old male. A large residual tumor (74.6 cm^3^) obtained after partial removal due to an impending brain hernia was treated with a marginal dose of 27 Gy in five fractions at an isodose of 56% (left). A significant tumor response with no adverse imaging effects was found four months after the administration of conformal radiotherapy (right). The patients’ left hemi-paresis disappeared, and the KPS improved from 60 to 70. **B**: Contrast-enhanced CT scans (MR images not available for the pace maker implant). Lung cancer brain metastasis in a 52-year-old female. A tumor in the thalamus (10.6 cm^3^) with perifocal edema was treated with a marginal dose of 31 Gy in five fractions at an isodose of 63% (left). A tumor response was observed seven months after the administration of conformal radiotherapy (right). The patients’ left hemiparesis was ameliorated, and the KPS improved 60 to 70 (walking with a stick 32 months after treatment). **C**: Gd-enhanced T1-weighted MR images. Breast cancer brain metastasis in a 70-year-old female. A large tumor in the parietal lobe (23.5 cm^3^) with perifocal edema was treated with a marginal dose of 35 Gy in eight fractions at an isodose of 57% (left). A tumor response was found two months after treatment, Gerstmann’s syndrome disappeared, and the KPS improved 60 to 70. Marginal recurrence was noted 20 months after the first treatment, and the recurrent lesion (1.7 cm^3^) was treated with a marginal dose of 20 Gy at an isodose of 69% in single-session radiosurgery (center). A tumor response with no adverse imaging effects was found 34 months after the first treatment (right).

**Figure 2:**
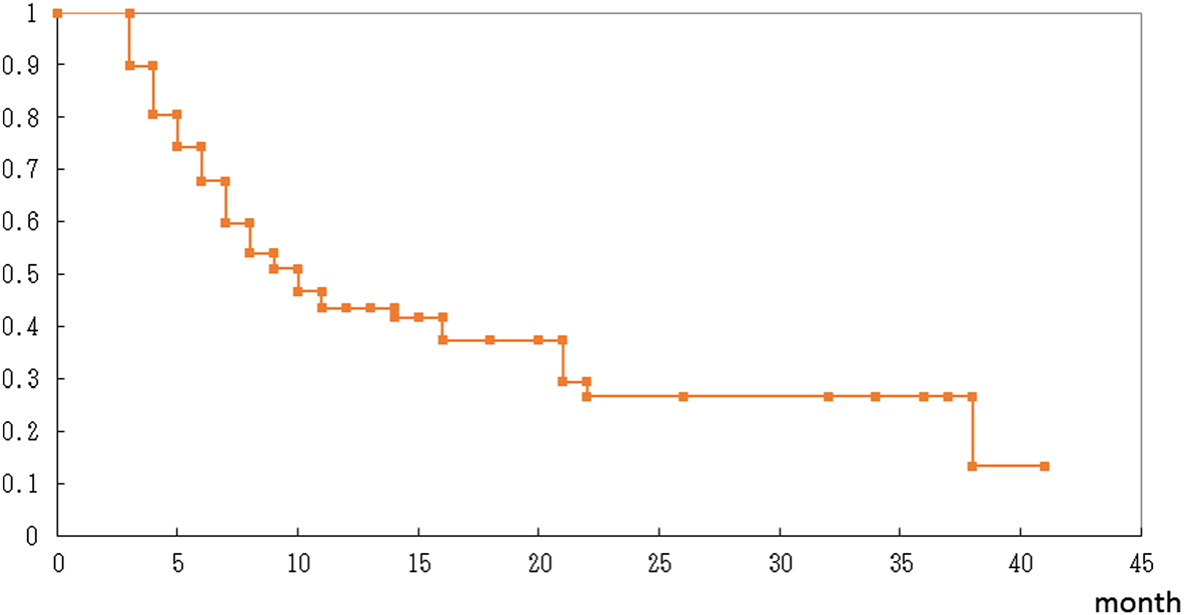
**Kaplan-Meier survival curves of the 88 patients with large brain metastases treated with optimal treatment.**

**Figure 3:**
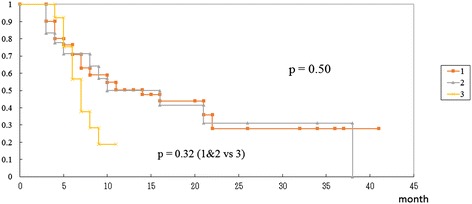
**Kaplan-Meier survival curves of the patients with large brain metastases in the three groups.** Group 1): patients with tumors measuring 10–19.9 cm^3^, group 2): patients with tumors measuring 20–29.9 cm^3^, group 3): patients with tumors measuring ≥30 cm^3^. No statistically significant differences were found between the groups.

All 92 lesions in the 88 patients were subjected to sequential imaging studies from one to 37 months (median, seven months) after treatment. All but five of the 92 lesions (three enlarged and two stable) showed tumor regression on follow-up images (Table 3, Figure 1). Six lesions exhibited marginal recurrence and required additional treatment. A second cycle of treatment was performed at the recurrent areas only, excluding the central areas treated with higher doses (Figure 1C). The local tumor control rate was 90.2%, with a median survival of nine months (Figure 2). There were no significant differences in the survival rates among the patients with tumors measuring 10–19.9 cm^3^, 20–29.9 cm^3^ or ≥30 cm^3^ after treatment (log-rank test: p =0.50, generalized Wilcoxon test for group 1&2 vs. 3: p =0.32) although the rate of survival was lower in the patients with tumors measuring ≥30 cm^3^ than in the other groups (Figure 3).

### Tumor recurrence

Marginal regrowth of the treated lesions occurred in six patients seven to 36 months after treatment; all tumors were located in the cerebral hemisphere (Table 4). The tumors were treated with a median prescribed isodose of 57% and median SDE of the maximum dose of 48.4 Gy. No significant factors were found in the univariate and multivariate analyses. However, the difference in the tumor volumes (10–19.9 cm^3^ vs 30–74.6 cm^3^) between the groups was significant (p =0.02), whereas the difference in the tumor location (cerebrum vs. others) was not (p =0.16). All tumors recurred more than six months after treatment, and the difference between the groups (followed for <6 months vs. ≥6 months) was found to be significant (p =0.001).

**Table 4:** **Characteristics of the six patients with recurrences after optimal hypofractionated conformal radiotherapy**

	**Median (range)**	**Univariate**	**Multivariate**		
		**p value**	**p value**	**HR**	**95% CI**
Age	68.5 (56–82)	0.17	0.20	1.09	0.95-1.25
Sex	M: 3, F: 3	0.91	0.29	0.23	0.01-3.63
Tumor location	Cerebrum (P: 2, F: 2, T: 1, O: 1)	0.85	0.75	1.13	0.53-2.39
Tumor volume (cm^3^)	18.0 (11.9-24.0)	0.69	0.69	1.04	0.85-1.29
Prescribed isodose (%)	57 (52–66)	0.97	0.73	0.93	0.63-1.37
Fraction number	5 (3–8)	0.89	0.73	0.82	0.27-2.49
SDE of the max. dose (Gy)	48.4 (36.8-54.3)	0.72	0.40	0.85	0.58-1.25
V14 (cm^3^)	4.9 (3.7-5.1)	0.78	0.90	1.07	0.35-3.32

HR: Hazard ratio, CI: Confidence interval, P: Parietal, F: Frontal, T: Temporal, O: Occipital.

### Adverse effects (brain edema)

Six patients who experienced recurrent symptoms and one patient who developed new symptoms due to extensive brain edema required osmo-steroid therapy. The symptoms and edema rapidly improved after the osmo-steroid therapy. Each of these patients received further oral administration of steroids at the outpatient clinic. Two patients who displayed recurrent symptoms and one patient who exhibited new symptoms due to perifocal edema required the oral administration of steroids at the outpatient clinic. These 10 patients showed both clinical and radiological deterioration one to 16 months after treatment. Two of these patients demonstrated newly developed brain edema, while the remaining eight patients presented with the extension of pre-existing brain edema that had been present prior to treatment. The median age of these patients was 69 years, which was older than that of the total population (Table 5). Each of these patients were treated with 5–10 fractions (median, 6). The V14 of the patients with brain edema ranged from 3.6 to 6.1 cm^3^. In the univariate analyses, age and the number of fractions were found to be significant factors for complications; however, only the number of fractions was found to be significant in the multivariate analyses. Differences between the groups were significant for each of the following factors: age (≥60, p = 0.02), number of fractions (≥5, p = 0.0006) and duration of edema (<6 months, p = 0.001). In contrast, the differences in tumor volume between the three groups were not significant, nor were the differences in the number of patients treated with or without osmo-steroid therapy during hypofractionation treatment.

**Table 5:** **Characteristics of the 10 patients with adverse effects after optimal hypofractionated conformal radiotherapy**

	**Median (range)**	**Univariate**	**Multivariate**		
		**p value (CH model)**	**p value**	**HR**	**95% CI**
Age	69 (59–84)	0.04* (0.03*)	0.14	1.05	0.99-1.11
Sex	M: 7, F: 3	0.15	0.08	0.26	0.06-1.18
Tumor location	Cerebrum: 9, cerebellum: 1	0.63	0.67	0.91	0.59-1.90
Tumor volume (cm^3^)	20.5 (10.0-32.9)	0.86	0.34	0.96	0.88-1.05
Prescribed isodose (%)	57 (51–65)	0.77	0.88	0.98	0.79-1.22
Fraction number	6 (5–10)	0.007* (0.004*)	0.007*	2.04	1.21-3.43
SDE of the max. dose (Gy)	48.0 (40.5-55.3)	0.50	0.51	0.94	0.77-1.13
V14 (cm^3^)	4.7 (3.6-6.1)	0.92	0.88	0.96	0.57-1.61

CH: Cox hazard, HR: Hazard ratio, CI: Confidence interval, *: Significant.

## Discussion

The prognosis of patients with brain metastases is related to the stage of the primary cancer, age and the KPS score [14,15]. The worst survival is seen in patients with a KPS of less than 70. In the present series, 38.6% of the patients had a KPS of less than 70 and 65.9% of the patients were 60 years of age or older. Although 78 patients (88.6%) were in RTOG-RPA class 2 or 3, the median survival of our patients was nine months. Furthermore, no statistically significant differences were found between the three groups of patients divided according to the tumor volume, although the survival rate and median KPS score were lowest in the largest group. Optimal hypofractionated conformal radiotherapy helps to increase the KPS, at least in patients with symptomatic lesions not directly affecting functional areas, and contributes to improving the prognosis of patients with large brain metastases, as previously reported [9].

Single-session radiosurgery is increasingly being used to treat brain metastases and has the benefits of a short treatment time, high tumor control rate and low risk of complications. However, large metastases are not suitable for treatment with single-session radiosurgery, as lower tumor control rates (85%) and higher complication rates (15%) than those for smaller metastases have been reported [2,16].

The optimal hypofractionation treatment in this series yielded a tumor control rate of 90.2% in the patients with large tumors. The median maximum dose (SDE) of 47.4 Gy at a median prescribed isodose of 57% in a median of five fractions appeared to be effective for most large brain metastases, in addition to a marginal dose of 20 Gy at the prescribed isodose of 50-60% for small tumors in single fraction radiosurgery. However, more than five fractions were used in cases involving large tumors measuring more than 20 cm^3^ or tumors associated with extensive brain edema in order to decrease the V14 values.

Consequently, tumor recurrence appeared in six patients more than six months after treatment, all of which originated from marginal areas treated with the prescribed isodose. Additional treatment was easily performed in these patients, because the volume of the recurrent tumors was not large, and the risk of radiation necrosis after the second treatment was assessed to be very low. As to risk factors for recurrence, a larger tumor volume, lower prescribed isodose and lower SDE of the maximum dose are potential candidates; however, no factors were found to be significant in the univariate or multivariate analyses in this study. The difference in tumor volume (10–19.9 cm^3^ vs 30–74.6 cm^3^) between the groups was significant; however, recurrence occurred in only four of 61 patients with smaller tumors. Although greater sample size is required for further statistical analyses, our findings indicate that optimal hypofractionated conformal radiotherapy is effective for treating large lesions with a low rate of recurrence.

Brain edema developed in 10 patients, mostly within six months. With respect to risk factors, age and the number of fractions were found to be significant in this series. Older patients’ brains with large metastases may be sensitive to irradiation stress or possibly exhibit greater vulnerability than the normal adult brain. More than five fractions were used to treat large tumor measuring more than 20 cm^3^, tumors associated with extensive edema or tumors located in critical areas. Tumors situated deep within the white matter have a tendency to cause brain edema, and factors related to the onset of edema may promote the development or re-appearance of brain edema. In contrast to that observed for radiation necrosis, most cases of brain edema developed several months after treatment, and all were reversible and recovered after either osmo-steroid therapy or oral steroid treatment. Therefore, optimal hypofractionated conformal radiotherapy is a safe treatment for patients with large metastases with high risk factors.

Conducting dose-volume prediction of complications is essential for providing optimal hypofractionation treatment. We previously reported a method for predicting radiation necrosis using a model that accounted for the SDE of the maximum dose and V14 [10]. Long-term experience with single-session radiosurgery has also confirmed the optimal treatment doses for individual pathologies in the brain. For example, a marginal dose of 12 Gy or 20 Gy is used to treat vestibular schwannoma or AVM, respectively. The long-term results have been shown to be satisfactory, with low rates of complications [17,18]. The optimal dose and fraction number for hypofractionation treatment for such pathologies may be determined by predicting the incidence of complications with respect to avoiding adverse effects on the surrounding brain.

In this prospective study, our findings demonstrated a rate of high tumor control and a low rate of complications in the treatment of large brain metastases with high risk factors. No patients with radiation necrosis required surgical resection during the median follow-up of seven to 41 months. Therefore, the administration of defined optimal hypofractionation treatment based on the dose-volume prediction of complications is effective and safe for the treatment of large lesions. However, the V14 may need to be further reduced to less than 3 cm^3^ when treating tumors situated deep within the white matter and/or exhibiting extensive perifocal edema, as the rate of recurrence of pre-existing edema was not low in the patients with a V14 of 3 cm^3^ or more after treatment in this series. Conformal radiotherapy with a prescribed isodose of 50% to 60% has the benefits of decreasing the V14 value due to a sharp fall-off in the dose distribution, as noted with single-session radiosurgery. Optimal treatment using a large fraction number also has the benefits of decreasing the V14 value and helps to avoid radiation necrosis, as demonstrated in this prospective study.

## Conclusion

This prospective study of optimal dose and fractionation treatment for large brain metastases with high risk factors showed satisfactory results for local tumor control and survival, with limited complications. Conducting dose-volume prediction of complications using the V14 is beneficial for preventing irreversible complications in the treatment of large brain metastases or other lesions in the brain.
